# Interdisciplinarity research based on NSFC-sponsored projects: A case study of mathematics in Chinese universities

**DOI:** 10.1371/journal.pone.0201577

**Published:** 2018-07-31

**Authors:** Zhi-Yi Shao, Yong-Ming Li, Fen Hui, Yang Zheng, Ying-Jie Guo

**Affiliations:** 1 School of Mathematics and Information Science, Shaanxi Normal University, Xi’an, China; 2 The Library, Shaanxi Normal University, Xi’an, China; 3 School of Computer Science, Shaanxi Normal University, Xi’an, China; 4 School of Foreign Languages, Shaanxi Normal University, Xi’an, China; Indiana University, UNITED STATES

## Abstract

We investigate the interdisciplinarity of mathematics based on an analysis of projects sponsored by the NSFC (National Natural Science Foundation of China). The motivation of this study lies in obtaining an efficient method to quantify the research interdisciplinarities, revealing the research interdisciplinarity patterns of mathematics discipline, giving insights for mathematics scholars to improve their research, and providing empirical supports for policy making. Our data set includes 6147 NSFC-sponsored projects implemented by 3225 mathematics professors in 177 Chinese universities with established mathematics departments. We propose the weighted-mean DIRD (diversity of individual research disciplines) to quantify interdisciplinarity. In addition, we introduce the matrix computation method, discover several properties of such a matrix, and make the computation cost significantly lower than the bitwise computation method. Finally, we develop an automatic DIRD computing system. The results indicate that mathematics professors at top normal universities in China exhibit strong interdisciplinarity; mathematics professors are most likely to conduct interdisciplinary research involving information science (research department), computer science (research area), computer application technology (research field), and power system bifurcation and chaos (research direction).

## Introduction

*Interdisciplinary research* integrates the perspectives, concepts, theories, tools, techniques, information, and/or data from different specialized knowledge or research practices [1]. Its purpose is to advance fundamental understanding and/or solve problems whose solutions are beyond the scope of a single research field [2]. because it is important to make breakthroughs and to achieve more relevant outcomes [3], China’s National Natural Science Foundation (NSFC) [4]and the USA’s National Science Foundation (NSF) [5] understand the importance of–and have established relevant polices to improve–interdisciplinary research. However, interdisciplinary research has also been associated with negative features, such as consistently lower funding success [6].

*Interdisciplinarity research* involves the study of interdisciplinarity among researchers, institutions, and research areas. These studies are conducted on the basis of assessments of published papers, research projects, patents and/or other factors. An analysis of 17.9 million papers spanning all scientific fields highlights the importance of interdisciplinarity to scientific impact [7]. Interdisciplinarity also helps improve the prediction accuracy of outstanding papers [8]. Interdisciplinary research helps improves specialized studies, while interdisciplinarity research helps decision makers to better understand the patterns of interdisciplinary research, which is critical for *scientific decision making*.

Based on *published papers*, researchers investigated interdisciplinarity in the following *research areas*: chemistry [9], physics [10], nanoscience and nanotechnology[11–13], biotechnology [14], information science and library science [15–18], humanities [19], bionanoscience [3, 20], Japanese rice research and technology development [21], biochemistry and molecular biology [22], medical science [23], and biotechnology [24]. The problem regarding studies based on published papers is the incomplete collection of bibliographic data, although this issue has recently been addressed from a technical perspective [25]. However, there have been only scant studies regarding interdisciplinarity as it pertains to *mathematics*.

Previous studies have addressed interdisciplinarity at research institutions. For example, Cassi et al. defined a framework for institutional interdisciplinarity analysis [26], and Gowanlock and Gazan investigated the interdisciplinarity of the NASA Astrobiology Institute at the University of Hawaii [27]. Jensen and Lutkouskaya investigated the interdisciplinarities of 600 laboratories of CNRS (Centre national de la recherche scientifique), which is the largest scientific organization in Europe [28]. However, few studies have focused on *normal universities*.

Interdisciplinarity studies based on an analysis of *sponsored projects* are limited, which may be a result of the difficulty in retrieving and analyzing scientific funding. The interdisciplinarities of social, behavioral, and economic sciences have been investigated under the auspices of the NSF [29]. Notably, NSFC data indicate that more than 59% of applicants change their application disciplines to pursue interdisciplinary funding applications [30]. Moreover, interdisciplinary big data studies in the US and China have been compared insofar as they relate to NSF- and NSFC-sponsored projects [31].

However, evaluating scientific funding plays an important role because it is critical for decision making. Inequality in scientific funding is increasing at an accelerated rate [32], and the “Matthew Effect” has been demonstrated to impact scientific research [33]. However, what are these effects and what should we do? If there were no previous studies of scientific funding, these answers would be difficult to ascertain. Notably, funding analyses are important to decision making [34]; thus, as a leading national government funding agency for basic science research, the NSFC has grown its annual budget from 80 million Yuan in 1986 to 24.8 billion Yuan in 2016. The NSFC funded 62.1% of Chinese research papers (equal to 11.5% of global academic output) in 2015 [35]. Thus, it will be of significant value to investigate the NSFC’s *funding patterns*.

Several prior studies refer to different research areas included in NSFC-sponsored projects, such as physical chemistry [36], microbiology [37–40], cardiovascular diseases [41], cancer research [42], health research [43], processing Chinese medical materials [44, 45], stroke [46], diabetic nephropathy [47], and wood science and technology [48]. However, few studies have examined NSFC-sponsored projects in the field of mathematics.

Other studies based on analysis of NSFC-sponsored projects are as follows. With respect to background, the global funding of economics research is substantially lower than the average funding level of social science research, and the Chinese funding ratio of economics ranks highest globally; however, the funding effect must be strengthened [49]. The funding ratio of social sciences is approximately 1/3 of the natural sciences. The “Matthew Effect” is a reality in Chinese funding distribution [50]. Interdisciplinarity research is beginning to be investigated using NSFC-sponsored projects [30], although the roles of the NSF and the NSFC in interdisciplinary big data research have previously been compared [31]. However, interdisciplinarity research based on analyses of NSFC-sponsored projects has only recently been initiated, and there are no related studies regarding mathematics to date.

Based on the sample of NSFC-sponsored projects of mathematics professors in Chinese normal universities, this paper aims to answer the following questions:

- *How should interdisciplinarity be quantified more exactly based on NSFC-sponsored projects*?
- *How should the interdisciplinarity score be computed more efficiently*?
- *What does the interdisciplinarity of mathematics research look like in China*?
- *What are the favorite interdisciplinary subjects of mathematics research*?

The remaining sections of this paper are organized as follows. The “Methodology” section presents the data collection and research methods. In the “Results and discussion” section, we analyze and visualize the results. Finally, in the “Conclusions” section, we summarize our work and address potential extensions for future research.

## Background

The National Natural Science Foundation of China (NSFC) is an institution directly under the state council of China and is the main financial supporting organization for natural science research in China, as its capital source is central government spending. The ISIS is the official internet-based information system launched by the NSFC, from which NSFC sponsored projects information of researchers can be retrieved.

Project 985 is a constructive project launched by the Chinese government to found world-class universities in the 21st century [50]. *“985” universities are top universities of China* which obtain substantially more government-funded money than other universities. Project 211 refers to the Chinese government’s endeavor to strengthen approximately 100 universities and key disciplinary areas to be the national priority for the 21st century [51]. *“211” universities are first-class universities of China*. A “985” university belongs to “211”, whereas the opposite relationship does not hold. *Normal universities are special and stand out in mathematics research in China*, and they play an important role in Chinese mathematics research as they are the cradles of mathematics teachers for middle schools, high schools, and even universities in China.

## Methodology

### Data collection

The target data are collected from the Internet-based Science Information System (ISIS) of the NSFC. The data collected from the ISIS refer to 3225 mathematics professors who work in 177 Chinese universities with an established mathematics department. These professors are ultimately responsible for 6147 projects, worth a total value of 2,444,849,368 Yuan. The data set can be divided into 3 kinds as demonstrated in Table 1. Data of these 3 kinds of universities comprise our data sample for this research. NSFC began in 1986 and the sponsored results of 2017 have not been published, thus the approval time of these projects collected spreads from 1986 to 2016. In order to demonstrate a comparison study, Chinese “985” universities, “211” universities, and normal universities are chosen. There are 39 “985” universities and 162 “211” universities. To avoiddouble counting, when saying “211” universities we mean those 77 “211” universities that don’t belong to project “985”. Finally, data of 37 “985” universities, 60 “211” universities, and 80 normal universities are collected, because data of universities such as National University of Defense Technology are strictly confidential and some other universities are unwilling to expose their data.

**Table 1:** Composition of the data set.

University type	Universities No.	Professors No.	Projects No.	Amounts
**985 universities**	37	1233	3381	¥1,484,560,668
**211 universities**	60	982	1472	¥517,476,200
**Normal universities**	80	1010	1294	¥442,812,500
**Total**	177	3225	6147	¥2,444,849,368

Two difficulties were encountered during data collection. The first issue involved ensuring professors’ identification. We objectively confirmed the job title information of the researchers independently based on the relevant institutions. The second issue was based on differences in the structures of the specialties of each university. In some universities, mathematics comprises an independent school, whereas at other universities, mathematics, computer science and other majors belong to the same school. Thus, it is difficult to ensure the research field of each professor. To solve this problem, we used the academic biography of the relevant professors to judge accurately. To store the data, we use Microsoft Excel 2016 to take full advantage of its various statistic functions. Each project record includes fields related to grant numbers, discipline application codes, project names, project principal, institution, approved amount, and approved period.

### Measuring interdisciplinarity

During our research, two important indicators, i.e., the discipline application codes (DAC) and diversity of individual research disciplines (DIRD), are used to quantify interdisciplinarity.

The DAC comprise a string typically with a length of at least 3 bits and at most 7 bits. When applying for a project, the researcher must choose a DAC for his/her application. The DAC reflects 4 levels of a research discipline: the research department, the research area, the research field and the research direction. For example, DAC “A040406” implies that the research belongs to the department of “mathematical science” (A), the research area is “Physics I” (A04), the research field is “Optics” (A0404), and the research direction is “Ultra intense and ultra-fast optical physics” (A040406).

The DIRD is a scientific indicator that was proposed by Jiang Wu [30] to reflect the diversity and interdisciplinarity of researchers, and it is computed as follows:

1. All DACs of a researcher sponsored by the NSFC are collected.
2. The DACs are transferred to DAC sets. An integrated DAC contains 4 levels; however, not all DACs contain all 4 levels, which complicates the computation of the DIRD. Thus, a DAC set is used to reflect all 4 levels, and the vacant levels are expressed using 0. Each DAC has one DAC set. For example, the DAC set of A040406 is {A, A04, A0404, A040406}, and the DAC set of A0404 is {A, A04, A0400, A040000}.
3. Compute *W*_*ij*_, an intermediate variable used to compute the DIRD, for each researcher. Let *N*_*p*_ denote the set of NSFC-sponsored projects that belong to researcher *p*, let *C*_*i*_ denote the DAC sets of the project *i*, and let |*C*_*i*_ ∩ *C*_*j*_| denote the number of *continuously equal* terms of *C*_*i*_ and *C*_*j*_. *W*_*ij*_ is thus computed as follows:
  $$W_{ij}=\{\begin{array}{c} \frac{1}{\left|C_{i}\cap C_{j}\right|+1},\;C_{i}\neq C_{j}\\ \;0,\;C_{i}=C_{j} \end{array},\;i,j\in N_{p}$$ (1)
4. Compute the intermediate variable *D*_*i*_, where *N*_*p*_ denotes the set of NSFC sponsored projects of the researcher *p* and n denotes the number of projects in this set. We can *later borrow* the *W*_*ij*_ matrix shown in the section of “Efficient computation of DIRD” to have a visually understanding *comparatively*, i.e., the numerator of Eq 2 is *similarly* as the sum of all the elements in *W*_*ij*_ matrix except those in the main diagonal.
  $$D_{i}=\frac{\sum_{j-1}^{n-1}W_{ij}}{n-1},\;j\neq i\;and\;i,j\in N_{p}$$ (2)
5. Compute the DIRD. The DIRD for the researcher *p* is computed as follows:
  $${DIRD}_{p}=\sum_{i=1}^{n}D_{i},\;i\in N_{p}$$ (3)

We refer to the DIRD proposed by Jiang Wu as *the traditional DIRD or the classic DIRD*. Our aim is to investigate the interdisciplinarities of mathematics professors in Chinese “985” universities, “211” universities, and normal universities, and our study defines the DIRD of a university as the summed weighted-mean DIRD (SWM_DIRD) of all related mathematics professors. The SWM_DIRD is an indicator that reflects the research interdisciplinarity of the professors in a university.

## Results and discussion

### Main results of this paper

The main results of this paper are as follows:

**R1:** A weighted-mean DIRD is proposed to more closely express interdisciplinarity.
**R2:** Several properties are identified during computation of the DIRD, which reduces the computation cost to $((1-\frac{C_{u}^{2}}{n^{2}})\times 100)\%$ of the traditional bitwise computation; n is the number of NSFC-sponsored projects that belong to the researcher and u denotes the number of unequal C_i_s.
**R3:** A DIRD automatic computing system is developed.
**R4:** The 985 universities which represent top universities of China exhibit stronger interdisciplinarity in mathematics research, compared with the 211 universities which represent the first-class universities and the normal universities which represent the general-level universities.
**R5:** Mathematics interacts most frequently with information science with respect to the research department, with computer science with respect to the research area, with computer application technology with respect to the research field, and with power system bifurcation and chaos with respect to the research direction.

### The design of DIRD index

#### The limitations of a traditional DIRD

To quantify the interdisciplinarity of individual researchers, Jiang Wu [30] proposed an indicator referred to as the DIRD (Diversity of Individual Research Disciplines). The DIRD reflects the interdisciplinarity of researchers on a scientific basis. However, its limitations arise in the investigation of the DIRD of the 5 top NSFC-sponsored research projects in 2015. These 5 projects respectively received funding of more than 70 million Yuan, whereas approximately 94.56% of projects respectively received funding of less than 1 million Yuan, as indicated in S1 Table. We are interested in the DIRD of the 5 top-sponsored researchers. The traditional DIRDs of the 5 top researchers are computed using Jiang Wu’s method and are presented in Table 2. The problem arises when we assess the DIRD scores of Researcher A and Researcher C. The interdisciplinarity reflected by the traditional DIRD is, to a certain extent, inconsistent with the data directly obtained from the DAC sets. The DAC sets of Researcher A and Researcher C are presented in S2 Table and S3 Table respectively. The different colors indicate the different DAC values. S2 Table indicates that Researcher A’s projects stretch across three departments: A (mathematical science), F (information science), and H (medical science). S3 Table indicates that Researcher C’s projects belong to only one department B (chemical science). Thus, the interdisciplinarity of Researcher A is substantially stronger than that of Researcher C, and the DIRD of Researcher A should be higher than that of Researcher C. However, the traditional DIRD of Researcher A is 5.2778, which is smaller than the value of 6.1736 for Researcher C. Thus, the interdisciplinarity is not exactly reflected. What are the reasons of this problem? We will illustrate in the following two points.

**Table 2:** Classic DIRD and weighted-mean DIRD.

Name	Researcher A	Researcher B	Researcher C	Researcher D	Researcher E
**Classic DIRD**	5.2778	5.0583	6.1736	7.58	0.6667
**Weighted-Mean DIRD**	0.1319	0.0176	0.0149	0.0146	0.0076

*The key point* is that the traditional DIRD does not reflect the different importance of the 4 DAC levels. The 4 DAC levels (scientific department, research area, research field, and research direction) should have different importance levels when interdisciplinarity is evaluated. Concretely, the importance of the 4 levels progressively decreases. Thus, to investigate the interdisciplinarity more precisely, we should assign corresponding weights for each level, which results in the weighted-mean DIRD.

*Another important point* is that the traditional DIRD score will fluctuate with the number of a scholar’s projects. As shown in Formula (3), the final procedure of computing DIRD is “${DIRD}_{p}=\sum_{i=1}^{n}D_{i},\;i\in N_{p}$”. Therefore, a bias arises. If a researcher owns more projects than another researcher, then his/her DIRD score may be higher than that of another. However, his/her real interdisciplinarity may not necessarily so stronger. The case mentioned above about Researcher C and Researcher A is just an example. Thus, in order to address this bias, another part of the scientific method “weighted mean” is adopted, i.e., the method of “mean”. Concretely, the final procedure of computing DIRD becomes “${DIRD}_{p}=\frac{1}{n}\sum_{i=1}^{n}D_{i},\;i\in N_{p}$”, where *n* represents the project number of the researcher *p*. As each *D*_*i*_ has already expressed the interdisciplinarity that the project *i* has gained in all the projects of researcher *p*, then the mean value of all the *D*_*i*_s can reduce the bias that caused by the number of projects.

Table 2 indicates the classic DIRD and the weighted-mean DIRD of the 5 top sponsored researchers. The Table indicates that the weighted-mean DIRD reflects interdisciplinarity in a manner that is closer to reality because the weighted-mean DIRD reflects the different importance of the 4 levels of the DAC. For example, the DIRD of Researcher A is the highest and the DIRD of Researcher E is the lowest, which is consistent with their DAC sets.

#### The weighted-mean DIRD

In the weighted-mean DIRD computing algorithm, the different importance of the 4 DAC levels in evaluating the interdisciplinarity is reflected by their different weights. Steps (1) and (2) in the algorithm are the same as those in the classic algorithm, as our key improvement starts from step (3). The algorithm runs as follows.

1. All DACs of a researcher sponsored by the NSFC are collected.
2. The DACs are transferred to DAC sets. The core of the computation of DIRD is about *w*_*ij*_, which represents the “dissimilarity” between project *i* and *j*. Given *C*_*i*_ and *C*_*j*_ and the definition of |*C*_*i*_ ∩ *C*_*j*_|, *W*_*ij*_ can be calculated without the introduction of Step 2. However, this step is retained, as it has something to do with the data processing procedure in our automatic computing system. The DAC is original placed in one cell of Excel, when DAC is transferred into DAC sets, each part of DAC sets will be put in an individual cell. Thus, we no longer need to introduce any pointers and variables. This will facilitate the computation of *W*_*ij*_ and thus make the automatic computing system less complex and much easier to be understood.
3. The intermediate variable, $W_{ij}=\frac{1}{weight\times |C_{i}\cap C_{j}|+1}$, is computed. There are 5 situations based on the number of continuously equal sectors between *C*_*i*_ and *C*_*j*_: 0 sectors, 1 sector, 2 sectors, 3 sectors, and 4 sectors. If no sectors are equal, the two projects belong to different scientific departments of the NSFC (for example, A01 and B01). In this case, we assign a weight of 0. If there is 1 equal sector, it must be the first level of the DAC, and the two projects belong to the same scientific departments (for example, A01 and A02). In this case, we assign the smallest weight of 1/4. If there are 2 equal sectors, they must be the same department and research area (for example, A0102 and A0103). In this case, we assign a weight of 2/4. If there are 3 continuously equal sectors, they must be in the same department, research area and research field (for example, A010203 and A010205). In this case, we assign a weight of 3/4. If all 4 sectors are equal (for example, A010203 and A010203), the two projects belong to the same research direction. In this case, we assign a weight of 1. *Intuitively*, *the importance of the 4 DAC levels should be as follows*: *department > research area > research field > research direction*. *However*, *the weights we assign are in an ascending order because these weights are placed in the denominator*. Assume *C*_*i*_ and *C*_*j*_ denote the DAC sets of projects *i* and j, respectively; if there is no equal sector, then *W*_*ij*_ = 1; if there is one equal sector, then $W_{ij}=\frac{1}{weight\times |C_{i}\cap C_{j}|+1}=\frac{1}{\frac{1}{4}\times 1+1}=\frac{4}{5}$. Moreover, |*C*_*i*_ ∩ *C*_*j*_| originally denotes the number of equal sectors of |*C*_*i*_ ∩ *C*_*j*_|; however, in this paper, we borrow the symbol of |*C*_*i*_ ∩ *C*_*j*_| to denote the *continuously equal* sectors of *C*_*i*_ and *C*_*j*_. The equal sectors that are not continuous with the previous sector are not counted because the four DAC levels have a subordinate relationship. For example, let *C*_*i*_ = {A,01,02,05} and *C*_*j*_ = {A,01,03,05} and then |*C*_*i*_ ∩ *C*_*j*_| = 2. The “05” is not counted because the research area “01” is under the same research department “A”, whereas the research directions “05” are under different research fields “02” and “03”, respectively. Thus, although the fourth sectors are equal, it makes no sense. If there are two equal sectors, then $W_{ij}=\frac{1}{weight\times |C_{i}\cap C_{j}|+1}=\frac{1}{\frac{2}{4}\times 2+1}=\frac{1}{2}$; if there are three equal sectors, then $W_{ij}=\frac{1}{weight\times |C_{i}\cap C_{j}|+1}=\frac{1}{\frac{3}{4}\times 3+1}=\frac{4}{13}$; if all four sectors are equal, then *W*_*ij*_ = 0. The lookup table is shown in Table 3.
4. The *W*_*ij*_ matrix is filled using the properties described in the following section.
5. The intermediate variable, $D_{i}=\frac{\frac{n_{0}}{n-1}\times 1+\frac{n_{1}}{n-1}\times \frac{4}{5}+\frac{n_{2}}{n-1}\times \frac{2}{4}+\frac{n_{3}}{n-1}\times \frac{4}{13}}{n-1}$, is computed, where *n* denotes the number of the researcher’s NSFC-sponsored projects and *n*_*i*_ denotes the counts of 1, 4/5, 1/2 or 4/13 in the *i*th line of the *W*_*ij*_ matrix.
6. The results are computed as $DIRD=\sum_{i=1}^{n}D_{i}$.

**Table 3:** Lookup table of *W*_*ij*_.

***C***_***i***_**∩*C***_***j***_	0	1	2	3	4
***Weight***	0	1/4	1/2	3/4	/
***W***_***ij***_	1	4/5	1/2	4/13	0

Following the previously described instructions, we develop *a weighted-mean DIRD automatic computing system* presented in S1 File. The input of the system is the DAC sets, and the output is the weighted-mean DIRD score of the NSFC-sponsored researcher.

#### Efficient computation of DIRD

**Fig 1:**
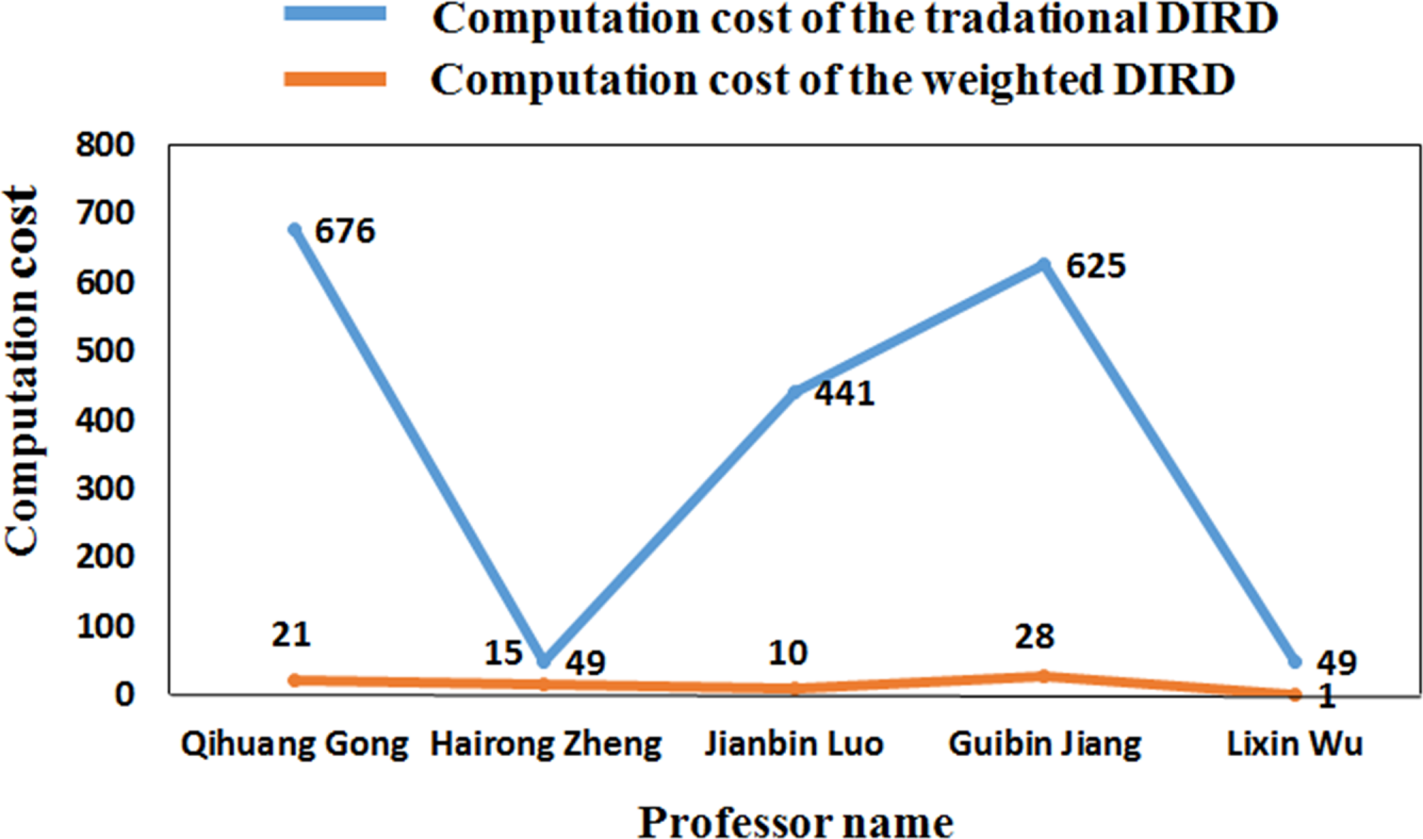
Reduction of the computation cost.

To improve computing efficiency, we introduce the *W*_*ij*_ matrix, a matrix that records the value of *W*_*ij*_. The *W*_*ij*_ matrix reduces the computation cost to a level of $((1-\frac{C_{u}^{2}}{n^{2}})\times 100)\%$ of the bitwise computation method, where *n* denotes the number of NSFC-sponsored projects that belong to a researcher and *u* denotes the number of unequal DACs (discipline application codes) that belong to the same researcher. The reduction is proportional to the DIRD of the researcher. A smaller DIRD is associated with a lower computation cost. Table 4 and Fig 1 present the computation costs of the bitwise method and the matrix method of the 5 top sponsored scholars in 2015. In Fig 1, the blue curve represents the comparisons performed using the traditional bitwise method of the DIRD computation, and the red curve represents the weighted-mean DIRD computation. For example, when computing Researcher D’s DIRD, the traditional method uses 676 comparisons, whereas the matrix method performs 21 comparisons.

**Table 4:** Reduction of the computation cost.

Name	Researcher D	Researcher A	Researcher B	Researcher C	Researcher E
**Classic cost**	676	49	441	625	49
**Reduced cost**	21	15	10	28	1
**Reduced/Classic rate**	3.11%	30.61%	2.27%	4.48%	2.04%

Let the *W*_*ij*_ matrix denote the matrix that records the value of *W*_*ij*_, let *N*_*p*_ denote the set of NSFC-sponsored projects that belong to researcher *p*, and let *C*_*i*_ denote the DAC set of project *i*. The previously described computing reduction is achieved by exploiting the following properties of the W_ij_ matrix.

1. the *W*_*ij*_ matrix is a symmetric matrix: *W*_*ij*_ = *W*_*ji*_, *i*,*j* ∈ *N*_*p*_;
2. the values on the diagonal of the *W*_*ij*_ matrix are 0s: *W*_*aa*_ = 0, *a* ∈ *N*_*p*_;
3. if *C*_*a*_ = *C*_*b*_, then *W*_*aj*_ = *W*_*bj*_, *a*,*b*,*j* ∈ *N*_*p*_;
4. if *C*_*a*_ = *C*_*b*_, then *W*_*ia*_ = *W*_*ib*_, *a*,*b*,*i* ∈ *N*_*p*_;
5. if *C*_*a*_ = *C*_*b*_, then *W*_*ab*_ = *W*_*ba*_ = 0,*a*,*b* ∈ *N*_*p*_;
6. if *C*_*i*_ = *C*_*j*_, then *W*_*ij*_ = 0; if |*C*_*i*_ ∩ *C*_*j*_| = 0, then *W*_*ij*_ = 1; and
7. By taking advantage of the lookup table to complete the *W*_*ij*_ matrix, we have only to perform $C_{u}^{2}=\frac{P_{u}^{2}}{2!}$ comparisons between *C*_*i*_*s*, where *u* denotes the number of unequal *C*_*i*_*s*. The computation cost is $\frac{C_{u}^{2}}{n^{2}}$ times the traditional bitwise method. The reduction rate compared with the traditional bitwise computation method is $((1-\frac{C_{u}^{2}}{n^{2}})\times 100)\%$, where *n* is the number of NSFC-sponsored projects of the corresponding researcher.

In the following analysis, we utilize the computation of Researcher B’s DIRD as *an example* to demonstrate how to compute the weighted-mean DIRD.

1. Transfer the DACs to the DAC sets as shown in Table 5.
  *C*_*i*_ is used to denote the DAC set of the *i*th DAC, and Researcher B’s DAC sets are presented in Table 6.
2. Identify the relationship between all *C*_*i*_s. As indicated in Table 7, the number of different *C*_*i*_ is 5, i.e., *C*_1_,*C*_2_,*C*_3_,*C*_4_,*C*_9_. Thus, we have only to perform $C_{5}^{2}=\frac{P_{5}^{2}}{2!}=10$ comparisons and take advantage of the lookup table to complete the *W*_*ij*_ matrix.
3. According to the relevant properties, only $C_{5}^{2}=\frac{P_{5}^{2}}{2!}=10$ comparisons must be performed to fix 10 *W*_*ij*_ values, as indicated in Table 8. In the traditionally bitwise DIRD computation, the DAC of the first grant is pairwise compared with the second grant, the third grant, and so forth. Using the bitwise method, we perform 21*21 = 441 comparisons to fix 441 *W*_*ij*_ values in the *W*_*ij*_ matrix. Using the new method, only 10 *W*_*ij*_ values must be fixed. Thus, the computation cost is 10/221 times the traditional method, yielding a reduction rate of [(1 − 10/221) × 100]% = 95.48%.
4. Fill the *W*_*ij*_ matrix presented in Table 9. Only the green values must be fixed by pairwise comparison, and all the other values may be directly filled using the properties.
5. Compute $D_{i}=\frac{\sum_{j=1}^{n-1}W_{ij}}{n-1},\;(j\neq i\;and\;i,j\in N_{p})$ and obtain $DIRD=\frac{1}{n}\sum_{i=1}^{n}D_{i}=0.0176,\;(i\in N_{p})$.

**Table 5:** Transfer the DACs to DAC sets.

No.	DAC	DAC set
1	E0505	{E, E05, E0505, E050500 }
2	E050501	{E, E05, E0505, E050501 }
3	E050503	{E, E05, E0505, E050503 }
4	E0512	{E, E05, E0512, E051200 }
5	E0505	{E, E05, E0505, E050500 }
6	E0512	{E, E05, E0512, E051200 }
7	E0505	{E, E05, E0505, E050500 }
8	E0512	{E, E05, E0512, E051200 }
9	E05	{E, E05, E0500, E050000 }
10	E050501	{E, E05, E0505, E050501 }
11	E0505	{E, E05, E0505, E050500 }
12	E05	{E, E05, E0500, E050000 }
13	E05	{E, E05, E0500, E050000 }
14	E05	{E, E05, E0500, E050000 }
15	E05	{E, E05, E0500, E050000 }
16	E05	{E, E05, E0500, E050000 }
17	E05	{E, E05, E0500, E050000 }
18	E05	{E, E05, E0500, E050000 }
19	E0505	{E, E05, E0505, E050500 }
20	E0505	{E, E05, E0505, E050500 }
21	E0505	{E, E05, E0505, E050500 }

**Table 6:** DAC sets of Researcher B.

C1 = {E, E05, E0505, E050500 }	C12 = {E, E05, E0500, E050000 }
C2 = {E, E05, E0505, E050501 }	C13 = {E, E05, E0500, E050000 }
C3 = {E, E05, E0505, E050503 }	C14 = {E, E05, E0500, E050000 }
C4 = {E, E05, E0512, E051200 }	C15 = {E, E05, E0500, E050000 }
C5 = {E, E05, E0505, E050500 }	C16 = {E, E05, E0500, E050000 }
C6 = {E, E05, E0512, E051200 }	C17 = {E, E05, E0500, E050000 }
C7 = {E, E05, E0505, E050500 }	C18 = {E, E05, E0500, E050000 }
C8 = {E, E05, E0512, E051200 }	C19 = {E, E05, E0505, E050500 }
C9 = {E, E05, E0500, E050000 }	C20 = {E, E05, E0505, E050500 }
C10 = {E, E05, E0505, E050501 }	C21 = {E, E05, E0505, E050500 }
C11 = {E, E05, E0505, E050500 }	

**Table 7:** Relationships among the DAC sets of Researcher B.

C1 = C5 = C7 = C11 = C19 = C20 = C21
C2 = C10
C3
C4 = C6 = C8
C9 = C12 = C13 = C14 = C15 = C16 = C17 = C18

**Table 8:** Matrix values that must be fixed.

W12	W13	W14	W19	W23	W24	W29	W34	W39	W49

**Table 9:** *W*_*ij*_ matrix.

	**1**	**2**	**3**	**4**	**5**	**6**	**7**	**8**	**9**	**10**	**11**	**12**	**13**	**14**	**15**	**16**	**17**	**18**	**19**	**20**	**21**
**1**	**0**	**4/13**	**4/13**	**1/2**	**0**	**1/2**	**0**	**1/2**	**1/2**	**4/13**	**0**	**1/2**	**1/2**	**1/2**	**1/2**	**1/2**	**1/2**	**1/2**	**0**	**0**	**0**
**2**	**4/13**	**0**	**4/13**	**1/2**	**4/13**	**1/2**	**4/13**	**1/2**	**1/2**	**0**	**4/13**	**1/2**	**1/2**	**1/2**	**1/2**	**1/2**	**1/2**	**1/2**	**4/13**	**4/13**	**4/13**
**3**	**4/13**	**4/13**	**0**	**1/2**	**4/13**	**1/2**	**4/13**	**1/2**	**1/2**	**4/13**	**4/13**	**1/2**	**1/2**	**1/2**	**1/2**	**1/2**	**1/2**	**1/2**	**4/13**	**4/13**	**4/13**
**4**	**1/2**	**1/2**	**1/2**	**0**	**1/2**	**0**	**1/2**	**0**	**1/2**	**1/2**	**1/2**	**1/2**	**1/2**	**1/2**	**1/2**	**1/2**	**1/2**	**1/2**	**1/2**	**1/2**	**1/2**
**5**	**0**	**4/13**	**4/13**	**1/2**	**0**	**1/2**	**0**	**1/2**	**1/2**	**4/13**	**0**	**1/2**	**1/2**	**1/2**	**1/2**	**1/2**	**1/2**	**1/2**	**0**	**0**	**0**
**6**	**1/2**	**1/2**	**1/2**	**0**	**1/2**	**0**	**1/2**	**0**	**1/2**	**1/2**	**1/2**	**1/2**	**1/2**	**1/2**	**1/2**	**1/2**	**1/2**	**1/2**	**1/2**	**1/2**	**1/2**
**7**	**0**	**4/13**	**4/13**	**1/2**	**0**	**1/2**	**0**	**1/2**	**1/2**	**4/13**	**0**	**1/2**	**1/2**	**1/2**	**1/2**	**1/2**	**1/2**	**1/2**	**0**	**0**	**0**
**8**	**1/2**	**1/2**	**1/2**	**0**	**1/2**	**0**	**1/2**	**0**	**1/2**	**1/2**	**1/2**	**1/2**	**1/2**	**1/2**	**1/2**	**1/2**	**1/2**	**1/2**	**1/2**	**1/2**	**1/2**
**9**	**1/2**	**1/2**	**1/2**	**1/2**	**1/2**	**1/2**	**1/2**	**1/2**	**0**	**1/2**	**1/2**	**0**	**0**	**0**	**0**	**0**	**0**	**0**	**1/2**	**1/2**	**1/2**
**10**	**4/13**	**0**	**4/13**	**1/2**	**4/13**	**1/2**	**4/13**	**1/2**	**1/2**	**0**	**4/13**	**1/2**	**1/2**	**1/2**	**1/2**	**1/2**	**1/2**	**1/2**	**4/13**	**4/13**	**4/13**
**11**	**0**	**4/13**	**4/13**	**1/2**	**0**	**1/2**	**0**	**1/2**	**1/2**	**4/13**	**0**	**1/2**	**1/2**	**1/2**	**1/2**	**1/2**	**1/2**	**1/2**	**0**	**0**	**0**
**12**	**1/2**	**1/2**	**1/2**	**1/2**	**1/2**	**1/2**	**1/2**	**1/2**	**0**	**1/2**	**1/2**	**0**	**0**	**0**	**0**	**0**	**0**	**0**	**1/2**	**1/2**	**1/2**
**13**	**1/2**	**1/2**	**1/2**	**1/2**	**1/2**	**1/2**	**1/2**	**1/2**	**0**	**1/2**	**1/2**	**0**	**0**	**0**	**0**	**0**	**0**	**0**	**1/2**	**1/2**	**1/2**
**14**	**1/2**	**1/2**	**1/2**	**1/2**	**1/2**	**1/2**	**1/2**	**1/2**	**0**	**1/2**	**1/2**	**0**	**0**	**0**	**0**	**0**	**0**	**0**	**1/2**	**1/2**	**1/2**
**15**	**1/2**	**1/2**	**1/2**	**1/2**	**1/2**	**1/2**	**1/2**	**1/2**	**0**	**1/2**	**1/2**	**0**	**0**	**0**	**0**	**0**	**0**	**0**	**1/2**	**1/2**	**1/2**
**16**	**1/2**	**1/2**	**1/2**	**1/2**	**1/2**	**1/2**	**1/2**	**1/2**	**0**	**1/2**	**1/2**	**0**	**0**	**0**	**0**	**0**	**0**	**0**	**1/2**	**1/2**	**1/2**
**17**	**1/2**	**1/2**	**1/2**	**1/2**	**1/2**	**1/2**	**1/2**	**1/2**	**0**	**1/2**	**1/2**	**0**	**0**	**0**	**0**	**0**	**0**	**0**	**1/2**	**1/2**	**1/2**
**18**	**1/2**	**1/2**	**1/2**	**1/2**	**1/2**	**1/2**	**1/2**	**1/2**	**0**	**1/2**	**1/2**	**0**	**0**	**0**	**0**	**0**	**0**	**0**	**1/2**	**1/2**	**1/2**
**19**	**0**	**4/13**	**4/13**	**1/2**	**0**	**1/2**	**0**	**1/2**	**1/2**	**4/13**	**0**	**1/2**	**1/2**	**1/2**	**1/2**	**1/2**	**1/2**	**1/2**	**0**	**0**	**0**
**20**	**0**	**4/13**	**4/13**	**1/2**	**0**	**1/2**	**0**	**1/2**	**1/2**	**4/13**	**0**	**1/2**	**1/2**	**1/2**	**1/2**	**1/2**	**1/2**	**1/2**	**0**	**0**	**0**
**21**	**0**	**4/13**	**4/13**	**1/2**	**0**	**1/2**	**0**	**1/2**	**1/2**	**4/13**	**0**	**1/2**	**1/2**	**1/2**	**1/2**	**1/2**	**1/2**	**1/2**	**0**	**0**	**0**

### A case study of mathematics in Chinese universities

#### Research process

**Fig 2:**
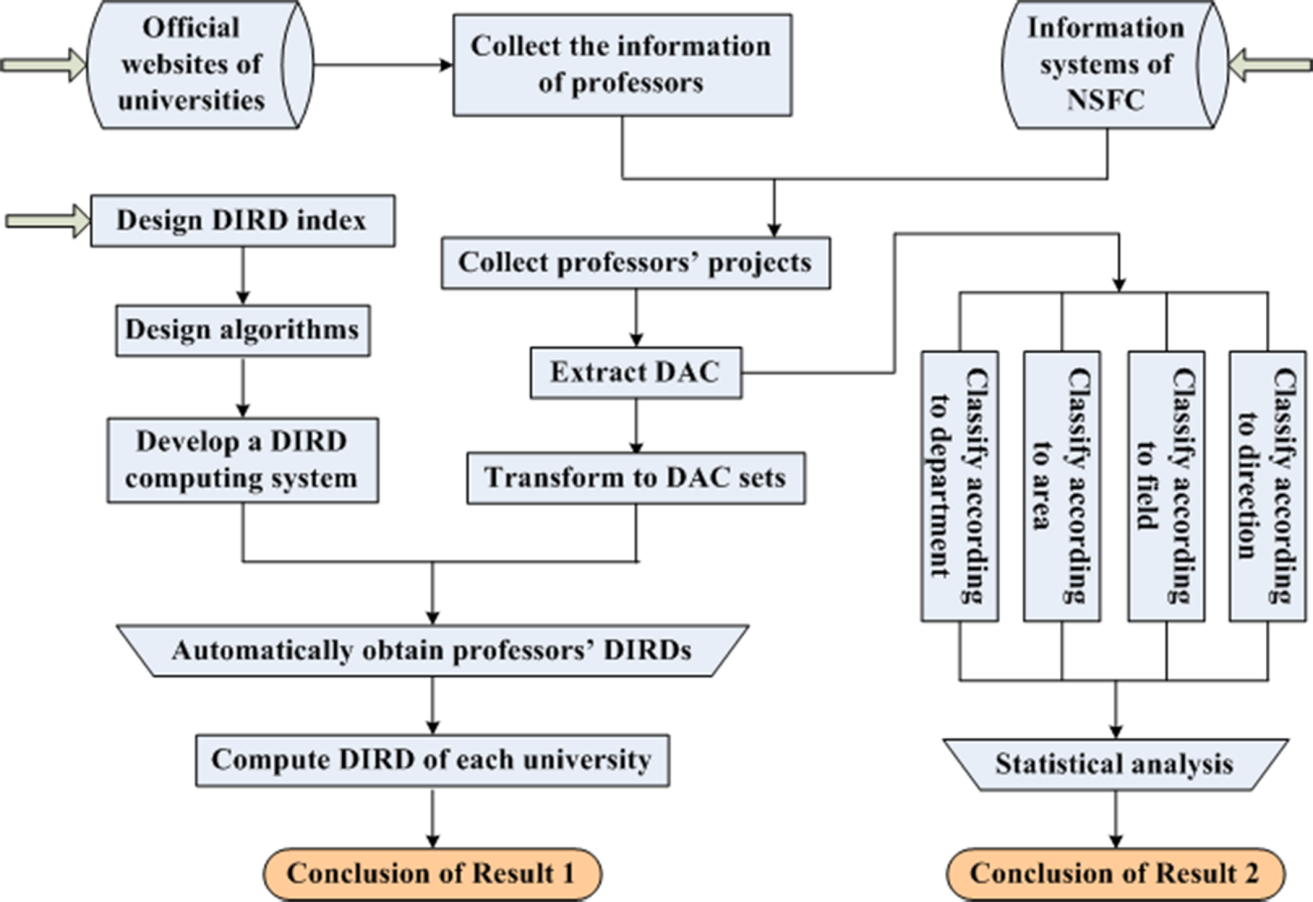
Research process.

In this section, a case study is performed to investigate the interdisciplinarity of mathematics professors at Chinese universities. Firstly, the DIRD of a university is defined as the summed weighted-mean DIRD of all its mathematics professors. Our research process can approximately be described as Fig 2. The first job is to collect the information of professors from the official website of each university. Finally, we obtain information of 3172 mathematics professors distributed in 177 Chinese universities. Then, the NSFC-sponsored projects of each professor are collected from the internet based science information system (ISIS) of NSFC. 6005 projects are collected in total. After the data cleaning, DACs of all these projects are extracted and transformed to DAC sets. Taking DAC sets of each professor as input, the weighted-mean DIRD computing system we have developed in advance can automatically output the weighted-mean DIRD of each professor. Then, according to the definition, DIRDs of all these 177 universities are computed. We finally obtain a comparison result of DIRDs of Chinese “985” universities, “211” universities and “normal” universities. From another point, after obtaining the DACs of professors’ projects, statistical analysis is performed to study which research department, research area, research field and research direction are mathematics professors’ favorite.

#### Interdisciplinarity investigation

**Fig 3:**
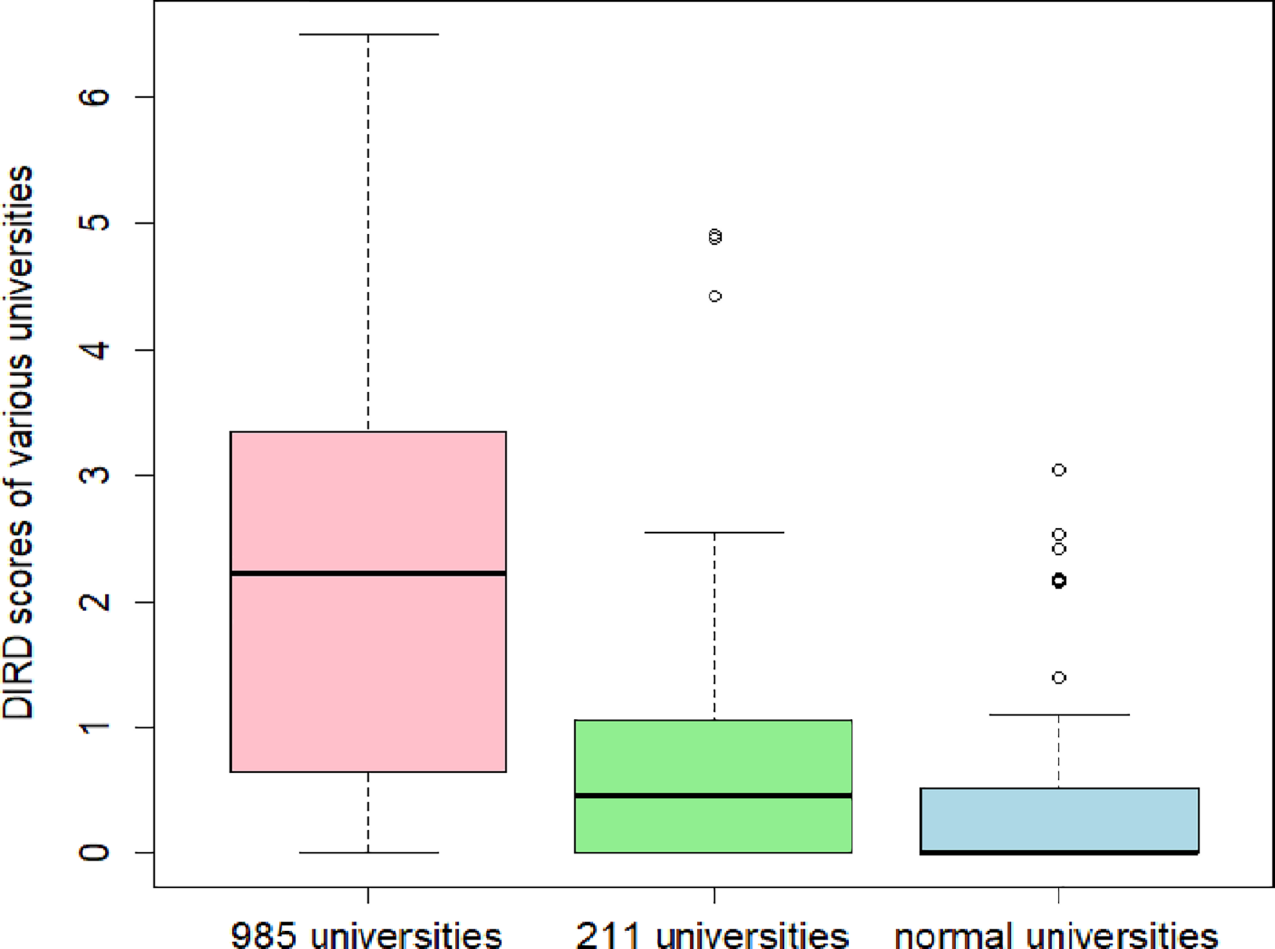
DIRD comparisons among “985”, “211” and “normal” universities.

DIRD reflects the degree of interdisciplinarity of universities. Mathematics professors at Chinese “985” universities, “211” universities and normal universities are investigated. Two normal universities, Beijing Normal University and East China Normal University, also belong to “985” project. There are still 7 normal universities which belong to “211” project. They are Shaanxi Normal University, Northeast Normal University, Central China Normal University, Nanjing Normal University, South China Normal University, Hunan Normal University, and Southwest University. “985” and “211” are reflections of the level of a university, whereas “normal” is a reflection of the property of a university. However, these three kinds of universities form the main strengths of mathematics research in China. Thus, they three are studied comparatively. Fig 3 demonstrates the final results. It is obviously that the interdisciplinarities of almost all the “985” universities are stronger than “211” universities, and the interdisciplinarities of all the “211” universities are stronger than “normal” universities. This indicates that mathematics professors at Chinese top universities show stronger research interdisciplinarities. Still, this can be confirmed by the average DIRD values of these three kinds of universities as shown in Table 10. Many more details are shown respectively in S2 File, S3 File, and S4 File.

**Table 10:** Average weighted-mean DIRD values of 3 kinds of universities.

"985" universities	"211" universities	"Normal" universities
2.281047213	0.814503877	0.346830689

The above result reflects interdisciplinarity of mathematics professors in Chinese universities. A lot of work still have to be done and researchers from many other fields have to participate in, if interdisciplinarity of all kinds of universities and of all disciplines need to be thoroughly explored.

Interdisciplinarity scores obtained in this study are indeed not very high, but this is easy to understand. Studies have shown that funding rate of interdisciplinary research is low [6]. Except having total confidence, most applicants demonstrate little interdisciplinarity although they perform highly interdisciplinary studies. For example, a mathematics researcher obtains a biological fund, and this means strong research interdisciplinarity, but this also means higher difficulty of being granted a fund. It is because that a mathematics researcher should submit his/her project to the research department of biology if he/she wants to obtain a biology fund. However, his/her biology fund application may not be so competitive as those of their biology competitors, and this will lead to his/her application failure. Thus, in order to obtain a fund first, researchers would rather apply in the research fields in which other researchers are familiar with him/her. This makes the fund easier to be granted. After having obtained the fund, the mathematics researcher could then perform interdisciplinary research with biology topics during his/her study. Such interdisciplinarity will be demonstrated by research papers, and this will be another research design of our study. Only when mathematics researchers are confident enough with their interdisciplinary research will they submit fund applications to the research department of biology. However, such confidence often comes along with their strong basis in biology research. Thus, the interdisciplinarity that DIRD score reflects is much stronger than the value itself can express.

However, DIRD provides a quantitative criteria for interdisciplinarity. It facilitates the study of changing rules of interdisciplinarity. DIRD acts as a ruler and interdisciplinarity acts as the height of a person. When there is no ruler, we can only intuitively say who is taller. However, having a ruler, we can not only say who is taller and how much taller, but also record his/her height in different periods of growth in order to explore his/her growth laws. *Thus*, *we need DIRD just as we need a ruler*.

#### Manifestation of mathematics professors’ interdisciplinary research

This section will investigate the manifestation of mathematics professors’ interdisciplinary research. The result will be demonstrated by answering the question that which research department, research area, research field and research direction are most closely involved during mathematics professors’ research. This job is performed by statistical analysis of all the 6147 NSFC-sponsored projects that mathematics professors ever undertook or are undertaking. Table 11 demonstrates that mathematics professors most enjoy to submit their applications to the research department of information science, and the complete information are shown in S4 Table. Computer science is their most favorite area when performing interdisciplinary research as shown in Table 12, and the complete information are shown in S5 Table. If focusing on research fields, as shown in Table 13, computer application technology is what mathematics professors most like to step in, and the complete information are shown in S6 Table. Although Table 14 shows that Power system bifurcation and chaos, which is a direction in the area of Mechanics, is mathematics professors’ favorite choice, almost all the other 9 directions in the table belong to the department of Information science, and the complete information are shown in S7 Table. Still, although these 9 directions do not all belong to the area of Computer science, they have very close relations with computer science. Thus, Mathematics is most closely involved with Computer science during mathematics professors’ interdisciplinary researches. This is also easy to understand. In the past in China, Computer science was a subdiscipline under Mathematics, and many universities had no independent school of computer science. Recently, most Chinese universities set up their own school of computer science, but a small number of universities still have no independent department of computer science. Even in universities that have independent school of computer science, some professors work at the same time in these two departments. Still, many famous computer scientists in China have an education background of Mathematics. *However*, *although Mathematics is most closely involved with Computer science*, *we are not sure whether the opposite relationship also holds in China*. *We will keep this question in mind and target it as one of our future studies*.

**Table 11:** Mathematics professors’ project number in different research departments.

Department code	Department name	Project number
F	Information science	550
G	Management science	92
E	Engineering and material science	40
C	Life Science	31
D	Earth Science	27
H	Medical science	13
B	Chemical science	1

**Table 12:** Mathematics professors' top 10 favorite research areas.

Research area code	Research area name	Project number
F02	Computer science	229
F03	Automation	203
F01	Electronics and information system	110
A02	Mechanics	103
G01	Management science and engineering	87
A05	Physics II	45
A04	Physics I	44
A06	Combination fund of NSFC and CAEP	21
D04	Geophysics and space physics	18
E09	Water science and marine engineering	16

**Table 13:** Mathematics professors' top 10 favorite research fields.

Research field code	Research field name	Project number
F0205	Computer application technology	100
F0301	Control theory and method	85
A0202	Dynamics and control	52
F0302	Systems science and systems engineering	46
F0104	Communication network	43
F0207	Information security	43
F0201	Basic theories of computer science	38
F0305	Artificial intelligence and knowledge engineering	38
G0103	Decision-making theory and method	29
A0501	Fundamental physics	28

**Table 14:** Mathematics professors' top 10 favorite research directions.

Direction code	Direction name	Project Number
A020202	Power system bifurcation and chaos	38
F020701	Cryptography	31
F030203	Theories and methods of complex system and complex network	31
F020501	Computer graphics	20
F010404	Mobile internet	19
F020507	Computer aided technology	17
F020509	Artificial intelligence application	17
F030108	Distributed parameter system control	15
F020104	Algorithm and time complexity	14
F030504	Data mining and machine learning	13

## Conclusions

This paper uses an analysis of NSFC-sponsored projects and investigates the interdisciplinarity of mathematics research in China in 37 “985” universities, 60 “211” universities, and 80 normal universities. We improve the precision of the indicator proposed by Wu et al. [30], develop a weighted-mean DIRD, improve computing efficiency, and develop an automatic DIRD computing system. We demonstrate that mathematics professors in Chinese top normal universities are characterized by stronger interdisciplinarity. In particular, mathematics professors are most likely to conduct interdisciplinary research in cooperation with information science (research department), computer science (research area), information security (research field), and cryptography (research direction). Our future work will aim to construct a test data set to further assess the scientific validity of the quantitative indicators and construct additional scientific indicators to quantify interdisciplinarity; aim to explore the comprehensive and potential research energy of Chinese universities; aim to explore the distribution rules of NSFC funds.

## Supporting information

S1 Table Number of sponsored projects in various ranges of sponsored amounts. (DOCX)

S2 Table DAC sets of Researcher A. (DOCX)

S3 Table DAC sets of Researcher C. (DOCX)

S4 Table Project number with respect to departments. (XLSX)

S5 Table Project number with respect to areas. (XLSX)

S6 Table Project number with respect to fields. (XLSX)

S7 Table Project number with respect to directions. (XLSX)

S1 File Weighted-mean DIRD computing system. (XLSX)

S2 File Data of 985 universities with weighted-mean DIRD. (XLSX)

S3 File Data of 211 universities with weighted-mean DIRD. (XLSX)

S4 File Data of normal universities with weighted-mean DIRD. (XLSX)
