# Supplementary material for: Interdisciplinarity research based on NSFC-sponsored projects: A case study of mathematics in Chinese universities
Source: PLoS One. 2018 Jul 31;13(7):e0201577. doi: 10.1371/journal.pone.0201577 (PMC6067728; doi:10.1371/journal.pone.0201577)
Supplement: S1 Table — (DOCX) [file pone.0201577.s001.docx]

**S1 Table.** **Number of sponsored projects in various ranges of sponsored amounts (million Yuan)**

| **Range** | (0, 1] | (1, 10] | (10, 20] | (20, 30] | (30, 40] | (40, 50] | (50, 60 ] | (60, 70] | (70, 80] |
| --- | --- | --- | --- | --- | --- | --- | --- | --- | --- |
| **Number** | 38584 | 2155 | 56 | 1 | 3 | 0 | 1 | 0 | 5 |
