# Supplementary material for: Interdisciplinarity research based on NSFC-sponsored projects: A case study of mathematics in Chinese universities
Source: PLoS One. 2018 Jul 31;13(7):e0201577. doi: 10.1371/journal.pone.0201577 (PMC6067728; doi:10.1371/journal.pone.0201577)
Supplement: S2 Table — (DOCX) [file pone.0201577.s002.docx]

**S2 Table.** **DAC sets of Researcher A**

| **No.** | **DAC** | **DAC set** |
| --- | --- | --- |
| 1 | H1805 | { H, H18, H1805, H180500 } |
| 2 | A040503 | { A, A04, A0405, A040503 } |
| 3 | A040503 | { A, A04, A0405, A040503 } |
| 4 | A040411 | { A, A04, A0404, A040411 } |
| 5 | H1825 | { H, H18, H1825, H182500 } |
| 6 | F010810 | { F, F01, F0108, F010810 } |
| 7 | A040501 | { A, A04, A0405, A040501 } |
