# Supplementary material for: Interdisciplinarity research based on NSFC-sponsored projects: A case study of mathematics in Chinese universities
Source: PLoS One. 2018 Jul 31;13(7):e0201577. doi: 10.1371/journal.pone.0201577 (PMC6067728; doi:10.1371/journal.pone.0201577)
Supplement: S3 Table — (DOCX) [file pone.0201577.s003.docx]

**S3 Table.** **DAC sets of Researcher C**

| **No.** | **DAC** | **DAC set** |
| --- | --- | --- |
| 1 | B0707 | { B, B07, B0707, B070700 } |
| 2 | B070704 | { B, B07, B0707, B070704 } |
| 3 | B07 | { B, B07, B0700, B070000 } |
| 4 | B07 | { B, B07, B0700, B070000 } |
| 5 | B0701 | { B, B07, B0701, B070100 } |
| 6 | B0702 | { B, B07, B0702, B070200 } |
| 7 | B07 | { B, B07, B0700, B070000 } |
| 8 | B070403 | { B, B07, B0704, B070403 } |
| 9 | B07 | { B, B07, B0700, B070000 } |
| 10 | B07 | { B, B07, B0700, B070000 } |
| 11 | B07 | { B, B07, B0700, B070000 } |
| 12 | B07 | { B, B07, B0700, B070000 } |
| 13 | B07 | { B, B07, B0700, B070000 } |
| 14 | B05 | { B, B05, B0500, B050000 } |
| 15 | B07 | { B, B07, B0700, B070000 } |
| 16 | B0701 | { B, B07, B0701, B070100 } |
| 17 | B07 | { B, B07, B0700, B070000 } |
| 18 | B07 | { B, B07, B0700, B070000 } |
| 19 | B07 | { B, B07, B0700, B070000 } |
| 20 | B07 | { B, B07, B0700, B070000 } |
| 21 | B0702 | { B, B07, B0702, B070200 } |
| 22 | B07 | { B, B07, B0700, B070000 } |
| 23 | B0701 | { B, B07, B0701, B070100 } |
| 24 | B0701 | { B, B07, B0701, B070100 } |
| 25 | B070101 | { B, B07, B0701, B070101 } |
